# Molecular Decoration of Ceramic Supports for Highly Effective Enzyme Immobilization—Material Approach

**DOI:** 10.3390/ma14010201

**Published:** 2021-01-03

**Authors:** Joanna Kujawa, Marta Głodek, Izabela Koter, Borys Ośmiałowski, Katarzyna Knozowska, Samer Al-Gharabli, Ludovic F. Dumée, Wojciech Kujawski

**Affiliations:** 1 Faculty of Chemistry, Nicolaus Copernicus University in Toruń, 7 Gagarina Street, 87-100 Toruń, Poland; joanna.kujawa@umk.pl (J.K.); marta.glodek1@umk.pl (M.G.); ikoter@umk.pl (I.K.); borys.osmialowski@umk.pl (B.O.); katkno@doktorant.umk.pl (K.K.); 2 Pharmaceutical and Chemical Engineering Department, German-Jordanian University, Amman 11180, Jordan; samer.gharabli@gju.edu.jo; 3 Institute for Frontier Materials, Deakin University, Geelong, Waurn Ponds, Victoria 3216, Australia; ludovic.dumee@ku.ac.ae; 4 Center for Membrane and Advanced Water Technology, Khalifa University, Abu Dhabi P.O. Box 127788, Saudi Arabia; 5 Department of Chemical Engineering, Khalifa University, Abu Dhabi P.O. Box 127788, Saudi Arabia

**Keywords:** surface modification, organic spacer, enzyme immobilization, *Candida antarctica* lipase B, ceramic membranes, alumina oxide

## Abstract

A highly effective method was developed to functionalize ceramic supports (Al_2_O_3_ powders and membranes) using newly synthesized spacer molecules. The functionalized materials were subsequently utilized for *Candida antarctica* lipase B enzyme immobilization. The objective is to systematically evaluate the impact of various spacer molecules grafted onto the alumina materials will affect both the immobilization of the enzymes and specific material surface properties, critical to enzymatic reactors performance. The enzyme loading was significantly improved for the supports modified with shorter spacer molecules, which possessed higher grafting effectiveness on the order of 90%. The specific enzyme activity was found to be much higher for samples functionalized with longer modifiers yielding excellent enantioselectivity >97%. However, the enantiomeric ratio of the immobilized lipase was slightly lower in the case of shorter spacer molecules.

## 1. Introduction

The effective and controllable modification process of organic or inorganic materials opens the gate for the unlimited number of novel surface modifications. Surface features may be tuned either by chemical or physical treatments, generating complex and functional, hybrid materials. During the design and engineering of bio-functional materials, such as those including enzymes or other biomolecules onto their surfaces, which may be generated through surface modifications, a balance between efficiency and reproductivity of the process as well as cost and simplicity should be established. Immobilization of enzymes can be accomplished either by physical adsorption or chemical immobilization, including covalent bonding and cross-linking [1,2]. In the case of physical adsorption, the enzymes conformation, critical to their reactivities and specific, is often well-maintained since adsorption is completed by either electrostatic interaction or van der Waals’ force, which do not denature biomalterials [3]. Nevertheless, such bonding is rather weak, thus leading to enzymes leaching from the surface of the supports during operation. Chemical processes via covalent bonding or cross-linking, however yield much stronger connections between both enzymes and substrate surfaces, supporting reusability and stability over cyclic testing [4,5]. One of the most commonly used modifiers for chemical enzyme immobilization is 3-aminopropyltriethoxysilane (APTES), a silane coupling agent. An advantage of APTES utilization is related to the ability to anchor aldehyde groups, opening routes towards catechol-based chemistries. Furthermore, APTES was recently used for the formation of the mussel-inspired coatings, which were found to be highly effective towards specific enzymes immobilization [6,7]. A cross-linking agent, such as glutaraldehyde (GA), often used to bond amino groups on the enzymes with the substrates surface. Such approaches however often lead to major disruptions in the enzyme structures and thus to conformational changes compared to the natural enzymes. APTES also possesses very short carbon chains which can severely limit both grafting and immobilization effectiveness. It is therefore necessary to generate new types of spacer molecules, which may support surface properties alterations while also stabilizing enzymes without denaturation.

Lipases (EC Number 3.1.1.3) are one of the most commonly used classes of enzymes in biocatalysis, which have been grafted onto a variety of substrates, showing very broad substrate specificities. Lipases catalyze the hydrolysis of triacylglycerols to diacylglycerol, monoacylglycerol, glycerol and free fatty acids. The reaction reverses under anhydrous conditions and the enzymes are able to synthesize new molecules by esterification, alcoholysis and transesterification. All reactions can be performed with high regio- and enantio-selectivity under mild reaction conditions. Lipase *Candida antarctica* type B from (CALB) is stable over a relatively broad pH range, especially in the alkaline pH range [8,9]. Lipase CALB has been used extensively in the resolution of racemic alcohols, amines, acids, and in the preparation of optically active compounds from meso substrates [10], as well as in polymer synthesis due to its high catalytic activity [11] sometimes supported by ionic liquids [9,12].

Apart from varied enzyme immobilization methods, numerous support materials with specific chemical and nanostructural properties have been studied. For instance, the immobilization of lipase CALB on silica aerogel [13], titanium and silicon dioxide [14], carbon nitride [15], and magnetic magnetite (iron oxide) nanoparticles [16] were reported. The feature of the supporting material (e.g., surface property, size, and morphology) have a substantial influence on the performance of the immobilized enzymes and amid various supporting materials, nanostructured ones have received growing interest since (i) providing a higher specific surface area for enzyme anchoring, and (ii) a high physical curvature allowing for a greater degree of freedom for the enzyme active sites location, thus minimizing lateral interactions between enzymes and self-digestion. The usage of nanostructured materials therefore supports greater enzyme activity and interactions with contaminants in solution [11,17,18,19,20,21,22]. A challenge of discrete nanoparticles for immobilization however lies with the dynamic nature of such slurries, leading over time to self-digestion through particle-particle contacts, using functionalized microporous supports such as membranes was found to limit losses in enzymic activity and thus long-term performance [23,24].

Compared to other nanoparticles, the unique properties of metal oxides such as TiO_2_, ZrO_2_ as well as Al_2_O_3_ nanoparticles, including their physical and chemical stabilities, low cyto-toxicity, and great coordination ability with amine and carboxyl groups, make them promising candidates for enzyme immobilization [1,25]. The application of metal oxide nanoparticles as supports for enzymes immobilization has been widely reported. Hu et al. [26] prepared a biosensor by immobilizing lyophilized horseradish peroxidase onto pristine titania oxide nanoparticles. The type of supporting material was found to be crucial on the electro-catalytic activity of the system showing much better performance for TiO_2_ compared to chitosan, nanoclay and gold nanoparticles as immobilization supports. Although promising performance were reported, these approaches were limited due to the physical adsorption of enzymes during the immobilization step. This step leads to enzyme detachment over time due to the weak bonding and to lower densities of enzymes attachment particularly when used in relatively aggressive environments, such as wastewaters and bio-reactors. The presence of hydroxyl groups on the surface of the metal oxide powders opens new chemical pathways for further chemical modifications of the surface of the particles supporting enhanced immobilization performance.

In the presented work, the main aim was focused on the selection of novel and more efficient ways of enzyme immobilization onto the surface of Al_2_O_3_ powders and porous ceramic membranes. Furthermore, due to the lack of suitable and highly effective linkers, a series of new spacer molecules were synthesized and used to decorate the substrates prior to enzymes the chemical grafting. Additionally, the systematical characterization of the materials, including the grafted and immobilized ones, was also performed to understand the impact of the surface modifications on the resulting enzymatic activity.

## 2. Materials and Methods

### 2.1. Materials

Ceramic materials, aluminum oxide (Al_2_O_3_) powders and ceramic ZrO_2_ membranes, were used as support materials. Al_2_O_3_ was purchased from Sigma-Aldrich (Warsaw, Poland). Ceramic planar membranes TAMI Industry (Nyons, France) with a diameter of 47 mm and a molecular weight cut off equal to 300 kDa were bought from Intermasz company (Września, Poland). The ceramic membranes possessed the selective layer made from zirconium oxide and support layer from the titanium oxide.

The following chemicals were purchased from Sigma-Aldrich (Warsaw, Poland), vinyl acetate (≥99%), glutaraldehyde (25% solution in water), tributyrin (98%), 4,9-dioxa-1,12-dodecanediamine (99%) and butylamine (99.5%). 1,4-diaminobutane 99% was provided by Acros Organics (Geel, Belgium), 1,10-diaminododecane 98% was bought from AmBeed (Arlington Heights, IL, USA), and 1,6-diaminohexane (98%) was purchased from Alfa Aesar (Kandel, Germany). (±)-1-phenylethanol was delivered by Fluka Chemie AG (Buchs, Switzerland)) Lipase from *Candida antarctica* type B (Lipozyme CALB L) was provided by Novozymes (Bagsværd, Denmark). Solvents: *n*-hexane, *iso*-propanol (HPLC grade), anhydrous dichloromethane, ethanol, and 3-isocyanatopropyltriethoxysilane (95%) were provided by abcr GmbH (Karlsruhe, Germany). Bio-Rad Protein Assay kit with a BSA standard was purchased from Sigma-Aldrich (Warsaw, Poland). All reagents were used as received without any additional purification.

### 2.2. Material Characterization of Ceramic Supports

Attenuated total reflection–Fourier transform infrared spectroscopy (ATR-FTIR) was used to characterize the surface modification effectiveness using a Bruker Vertex 80v (Bruker Optick GmbH, Ettlingen, Germany). A total of 512 scans were collected with a resolution of 4 cm^−1^.

Morphological properties of the samples were characterized by scanning electron microscopy (SEM) (Phenom ProX Desktop SEM, ThermoFisher Scientific, Gloucester, UK) with a back-scattered electron (BSE) detector. Samples were sputtered with a gold nanolayer (Au thickness layer—5 nm) to enhance the sample conductivity. The images were collected under 10 kV of acceleration; however, the mapping with EDX X-ray dispersion detector was performed at 15 kV.

The hybrid goniometric measurements were done with a goniometer equipped with 3D topography module for the roughness corrected contact angles determination (Attention Theta from Biolin Scientific, Gothenburg, Sweden). The profile was achieved by the implementation of fringe projection phase-shifting operated by 3D topography module. Subsequently, the same area of the sample was analyzed by depositing the drop of testing liquid and determining the contact angle. For data acquisition and analysis, One Attension software was utilized. The advantage of the method is the possibility to determine the apparent and intrinsic contact angle (corrected by roughness). The details related to the fringe projection phase-shifting are presented in Supporting Information (Figures S1 and S2). DI water (18 MΩ cm) was used as a testing liquid during the goniometric measurements with a constant volume of 3 μL and equilibration time of 5 s. CA was determined with ± 0.5° accuracy at room temperature. From the analysis of the registered data, it was possible to determine values of apparent contact angle (CA) and contact angle corrected by the roughness (CA_cor_). The roughness was expressed by the S_q_ root mean square (RMS) roughness and S_a_ arithmetic average, both factors are roughness parameters defines according to the ISO 25178 standard. The goniometric method allowed also to calculate the work of adhesion (W_adh_) and spreading pressure (S) [27,28,29,30].

Thermogravimetric analysis (TGA), differential thermal analysis (DTA), and derivative thermogravimetry (DTG) were performed across a temperature range of 25 to 1100 °C, with a heating rate of 20 °C/min under nitrogen, using Jupiter STA 449 F5 (Netzsch, Germany). DTA provided data related to changes in the energy of materials, in terms of enthalpy of the reaction and specific heat capacity, while TGA was used to determine the effectiveness of functionalization according to a method described elsewhere [31,32].

High-resolution transmission electron microscopy (HR-TEM) was performed using Tecnai G2 F20 X-Twin (FEI Europe, B.V., Eindhoven, The Netherlands), applying the accelerating voltage of 200 kV. TEM with HAADF high-angle annular dark-field and bright field imaging was implemented for the characterization of the modification process.

Nuclear magnetic resonance (NMR) measurements (^1^H and ^13^C) were performed using Bruker Avance 700 MHz (Bruker, Rheinstetten, Germany) using CDCl_3_ as a solvent and TMS as a reference for ^1^H and ^13^C. All spectra were recorded at room temperature.

Litesizer 500 (Anton Paar, Austria) was used for determining the particle size and zeta potential. The stock dispersion was performed in deionized water (18 MΩ·cm) at a concentration of 1.5 mg/mL. Prior to the analysis, samples were dispersed with ultrasounds for 5 min and then directly diluted to the final concentration (100 μg/mL) and analyzed. DLS (dynamic light scattering) and zeta potential were measured at 25 °C in deionized water (pH 5.8). The general-purpose model was used due to the low conductivity of the samples. Smoluchowski’s approximation has been used to convert the electrophoretic mobility into zeta potential [33].

### 2.3. The Synthesis of Surface Modifiers

The Al_2_O_3_ powders and ZrO_2_ membranes were modified using the same conditions. Modifiers **5a**–**d** were obtained in the reaction of 3-isocyanatopropyltriethoxysilane **1** (1 eq—*equivalent*) and diamine **4a**–**d** (4 eq) in anhydrous dichloromethane (DCM) according to the slightly modified procedure described elsewhere [34,35] and used in the grafting process without isolation in their pure form. The large excess of the diamine was used to avoid the risk of di-substitution of diamines by the isocyanate leading to bis-urea. However, to confirm that the reaction proceeds smoothly, the monoamine was used. The test reaction was focused on obtaining urea **3**. The product of reaction 3-isocyanatopropyltriethoxysilane **1** and amino-butane **2** (Figure S3) was isolated and characterized by ^1^H and ^13^C NMR (Figures S4 and S5) and IR (Figure S6).

3-Isocyanatopropyltriethoxysilane **1** (247.0 mg, 1.0 mmol) was added to solution of amino-butane **2** (73.0 mg, 1.0 mmol) in anhydrous dichloromethane (20 mL). Then the mixture was stirred overnight under a nitrogen atmosphere at room temperature. The solvent was evaporated to give compound **3** as yellow pale oil in quantitative yield. ^1^H NMR (700.27 MHz, CDCl_3_): *δ* = 4.75 (bs, 1H, NH), 4.60 (bs, 1H, NH), 3.81–3.77 (m, 6H, CH_2_CH_3_), 3.14–3.11 (m, 4H), 1.61–1.57 (m, 2H), 1.47–1.43 (m, 2H), 1.35–1.29 (m, 2H), 1.23–1.18 (m, 9H, CH_2_CH_3_ + CH_3_CH_2_OH), 0.90 (t, 3H, *J* = 7.5 Hz, CH_3_), 0.63-0.60 (m, 2H); ^13^C NMR (176.05, CDCl_3_): δ = 158.85, 58.48, 42.94, 40.21, 32.50, 23.74, 20.12, 18.33, 13.86, 7.68; IR: 3328, 2958, 2930, 2871, 1623, 1564, 1459, 1441, 1254, 1041 cm^−1^. It is worth to mention that a small amount of the (EtO)_3_Si- moiety decomposed giving the ethanol molecule visible in the ^1^H and ^13^C (58.28 and 18.41 ppm) spectra [36]. Thus, subsequent reactions with the use of diamines were directly used in the grafting process to limit the number of stages taken in the whole process.

### 2.4. Grafting Procedure

The grafting process of the Al_2_O_3_ powders and ceramic ZrO_2_ membranes was performed by applying a multistep procedure, presented elsewhere [31,32]. The powder of the oxide and membranes were dried at 50 °C for 6 h and stored in a desiccator overnight to maintain constant temperature and humidity conditions.

Prior to the grafting, the modifier **5a**–**d** was synthesized following the scheme presented in Figure S7. To the solution of diamine **4a**–**d** (4 eq, in anhydrous DCM) the 3-isocyanatopropyltriethoxysilane **1** (1eq) was added (30 mL, anhydrous DCM) at −20 °C. The ice bath was removed, and the mixture was stirred overnight under a nitrogen atmosphere at room temperature. The ratio of modifiers (in mmol) to a mass of Al_2_O_3_ (in g) is expressed as Q_MeOx_ parameter (Equation (1)). 15 mL of DCM solution of the modifier (0.05 M) was added to 1.0 g of dried Al_2_O_3_ powder. The reaction vessel was closed tightly and mixed for 72 h at room temperature on a roller mixer (Stuart SRT6D, Bibby Scientific Limited, UK) to provide complete contact of samples with the modifier. After that, the grafted Al_2_O_3_ was washed with dichloromethane, aqueous HCl (pH = 2–3), solution of NaHCO_3_, distilled water (five times in portions of 10 mL), and dried over 24 h at 50 °C. Modified Al_2_O_3_ powders were then analyzed by TGA, FT-IR, HR-TEM techniques.

(1) $$Q_{\mathrm{MeOx}}=\frac{\mathrm{amount}\;\mathrm{of}\;\mathrm{modifier}\;}{{\mathrm{Al}}_{2}O_{3}\;\mathrm{mass}}\left[\frac{\mathrm{mmol}}{g}\right]$$

**Figure 1:**
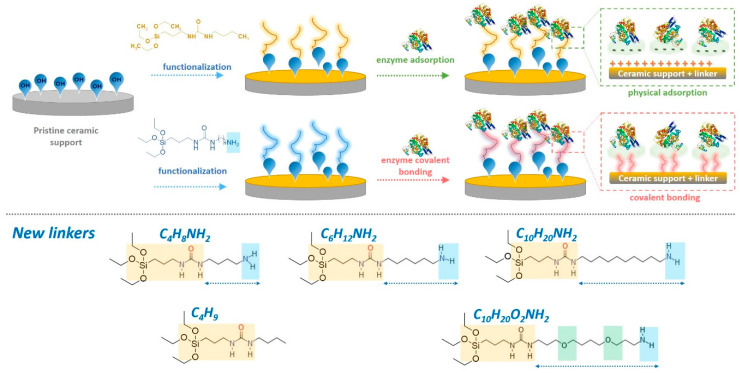
Scheme of the ZrO_2_ ceramic membrane functionalization by different modifiers and enzyme modifications (upper part) via physically adsorbed enzyme and covalently bound enzyme. Presentation of newly synthesized linkers (lower part).

In the case of the grafting process (Figure 1) of ZrO_2_ membranes, the concentration and the volume of the solution of modifiers were respectively 0.05 M and 60 mL.

### 2.5. Physical Adsorption of Lipase

Physical adsorption of lipase on the powder surface was accomplished according to the following procedure [37,38]. CALB L (2.0 mL, ~10 mg protein) was dissolved in 25 mL of phosphate buffer, 10 mM, pH = 7.0. The pH range presented by the supplier (Novozymes) as an optimal one was between 5 and 9. For that reason, a pH of 7 was selected. The powdered support (0.2 g) was placed in a 50 mm column attached to the isocratic HPLC pump, and the lipase solution was pumped through the column for 18 h at 20 °C with a flow rate 0.2 mL/min. The immobilized lipase was filtered off, washed with propan-2-ol and *n*-hexane, dried for 4 h at room temperature and stored at 4 °C.

In the case of the physical adsorption of lipase on the membrane, the samples with MWCO-50kDa were chosen. The sample was placed in a cross-flow reactor (Figure S8). Lipase solution (~10 mg protein) in 50 mL 0.01 M phosphate buffer pH 7.0 was circulating for 18 h at 20 °C. After immobilization the sample was washed with propan-2-ol and *n*-hexane, dried for 4 h at room temperature, and stored at 4 °C.

The amount of immobilized lipase was calculated as a difference of mass of protein in the enzyme solution before and after immobilization. Protein concentration was determined spectrophotometrically by Bradford method using BSA as a standard.

### 2.6. Covalent Immobilization of Lipase

The enzymes were attached onto the functionalized support via glutaraldehyde (GA) (Figure S9). Aldehyde groups reacted with amino groups of enzymes, thus supporting the covalent immobilization of lipase. Grafted alumina powders and ceramic membranes were treated with glutaraldehyde (GA) solution (1% *v*/*v* in phosphate buffer pH 7. 10 mM) for 2 h at room temperature. The powders and membranes were rinsed several times with deionized water to remove the residual GA. Lipozyme (CALB L) solution in phosphate buffer pH 7 (10 mM) was passed through the membrane for 10 h at 12 °C with an axial velocity of ca. 1·10^−4^ m s^−1^. The membrane was subsequently washed with DI water and stored at 4 °C until use. The GA-modified powders were packed into the small steel column and lipozyme (CALB L) solution in phosphate buffer pH 7 (10 mM) was circulating for 10 h at 12 °C. The efficiency of the lipase load on the support was analyzed by the Bradford assay using BSA as a standard [39]. The amount of immobilized lipase was defined as protein quantity and was calculated by subtracting the amount of free lipase in the solution from the total amount of the lipase used for immobilization.

### 2.7. Transesterification of (±)-1-Phenylethanol

Transesterification of racemic alcohol was carried out in an organic solvent batch reaction system consisted of 4 mL of *n*-hexane as solvent dissolving 0.026 g (0.2 mmol) of (*R,S*)-1-phenylethanol, 0.034 g (0.4 mmol) of vinyl acetate. Free enzyme solution (0.01 mL) or immobilized lipase powder (0.01 g) was added. The reaction mixture was incubated at 30 °C with constant stirring (150 rpm) under magnetic stirrer. Aliquots were withdrawn at specified time intervals from the reaction mixture and analyzed by chiral HPLC to evaluate the percentage of conversion and the enantiomeric excess of reactant and products. Substrate and product peak areas were compared, and the sum of the two was considered as 100%. The enantiomeric ratio (E-value) was calculated from the enantiomeric excess of the product (*ee_p_*), and the conversion degree (c) according to the method described by Chen et al. [40]. HPLC conditions: Daicel Chiralcel OD-H (250 × 4.6 mm, 5 µm) column with a Chiralcel OD precolumn; n-hexane/propan-2-ol 90/10 (*v*/*v*), 1 mL/min, Shimadzu SPD-10A VP UV-Vis detector (254 nm) (Shimadzu Europe, Duisburg, Germany).

### 2.8. Transesterification of (±)-1-Phenylethanol in the Membrane Reactor

A solution of racemic (*R,S*)-1-Phenylethanol (5 mmol) and vinyl acetate (2-fold molar excess) in *n*-hexane (50 mL) was circulating in a cross-flow membrane reactor system with an axial velocity of ca. 1·10^−5^ m s^−1^. Aliquots were periodically withdrawn and analyzed by HPLC to calculate conversion and enantioselectivity. Flat-sheet membranes (300 kDa) with the active surface area of 14.5 cm^2^ were used during the measurements. The operating temperature was equal to 30 °C. A cross-flow, home-made module made from stainless steel and Teflon was used.

## 3. Results and Discussion

### 3.1. Grafting Process of Al_2_O_3_ Powders and ZrO_2_ Membranes—Grafting Effectiveness

#### 3.1.1. Al_2_O_3_ Powders

In order to understand the grafting process across the surface of the membranes, as a first step, the model grafting reactions were performed onto the surface of Al_2_O_3_ powders with the newly synthesized spacer molecules (Figure 1). The selection of powders prior to the membrane modification, gave also better opportunity to characterize them with the utilization of broader range of analytical methods. Due to the fact that the amount of available hydroxyl groups onto the material surface is one of the most important factors, aluminum oxide (Al_2_O_3_) possessing the highest level of OH groups was selected.

**Figure 2:**
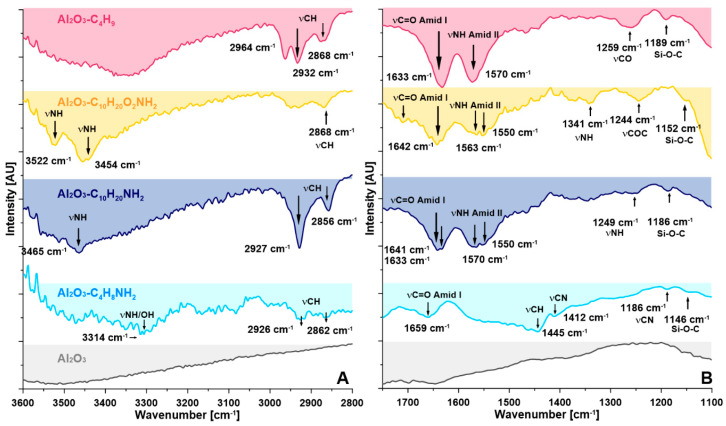
ATR-FTIR spectra of pristine and modified samples. (**A**) In the range of 3600–2800 cm^−1^; (**B**) at the range of 1750–1100 cm^−1^.

As seen across the ATR-FTIR spectra (Figure 2, Figures S10 and S11) the functionalization of the synthesized spacer molecules onto the surface of Al_2_O_3_ powder was achieved. The covalent link of the spacer molecules to the surface was characterized by the presence of specific vibration bands of alkylsilanes in the dactyloscopic region 1300–900 cm^−1^ (Figure 2 and Figure S10); 1190–1100 cm^−1^ (–Si–O–Si– stretching mode) (Figure 2), and 800–700 cm^−1^ (–Si–O–Si–) (Figure S11). The absence of characteristic peaks at 950–900 cm^−1^ (–Si–OH, –Si–OEt stretching mode) (Figure S11) confirmed the lack of unused –OEt groups, pendant on the non-grafted molecules and that is therefore an indication of the very high grafting efficiency of the proposed process [41,42,43]. In the case of the two modifiers (i.e., C_4_H_8_NH_2_, and C_10_H_20_NH_2_) with a different length of alkyl chain length possessing four and ten carbon atoms respectively, quite similar ATR-FTIR spectra were acquired. Bands located in the range of 2850–3030 cm^−1^ showed asymmetric and symmetric CH_2_ stretching vibrations of the alkyl chain of the modifiers. The bands at 1442 and 1400 cm^−1^ are related to asymmetric vibration bands of CH_3_ and in-plane deformation of CH_2_. The characteristic bands of NH (γ_N-H_ 3314 cm^−1^; δ _N-H_ 1590 cm^−1^, Amide II; δ _N-H_ 1323 cm^−1^, Amide III) and C=O groups (γ_C=O_ 1640–1659 cm^−1^, Amide I) were also found across all samples. Furthermore, bands at around 3520 cm^-1^ showed the presence of available amine terminal groups. In the case of modification with C_10_H_20_O_2_NH_2_ additional band at 1244 cm^−1^ was associated with a –C–O–C– bonding and –C–O– stretching bands (1117 cm^−1^) available in the modifier chain. However, the band at ~1470 cm^−1^ was related to the rocking vibration of –CH. Finally, for the modifier C_4_H_9_ possessing the methyl group as a terminal one, the bands at ~3520 cm^−1^ were not present. Clear and intense bands at a wavenumber of γ_C-H_: 2964, 2932, 2868 cm^−1^ were also observed for these spacer molecules [43].

**Figure 3:**
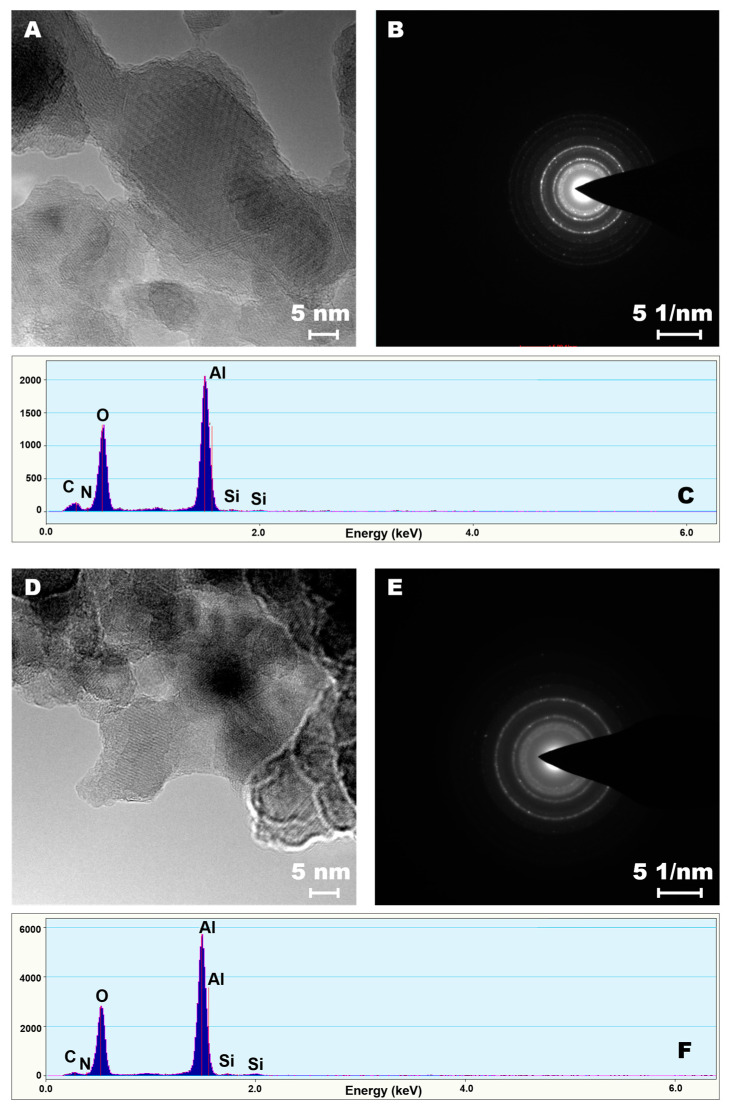
HR-TEM—bright field (**A**,**D**), selected electron area diffraction (SEAD) images (**B**,**E**) and EDX spectra (**C**,**F**). A, B, C—sample modified with C_4_H_8_NH_2_, D, E, F —sample functionalized with C_4_H_8_NH_2_ and enzymes.

The HR-TEM analysis, demonstrated that the entire samples were modified uniformly (Figure 3). To prove the highly efficient modification, bright field imaging and SEAD (selected electron area diffraction) analyses were performed. Although no major differences were observed on the transmission images, the changes on the diffraction patterns were noticeable. Upon functionalization of the alumina materials, an increase in counts for carbon and new peaks for silicon and nitrogen atoms densities were observed (Figure 3 and Figure S12). Furthermore, after enzyme immobilization, a substantial increase in oxygen and carbon atoms densities was noticed. The samples modified with other molecules are presented in Supporting Information file for the readers’ interest (Figures S12 and S13).

The quantitative analysis of the functionalization efficiency (E_f_) was performed with a TGA method [31,32] by determining of the number of hydroxyl groups available before and after modification (Equations (2) and (3)).

(2) $$n_{OH}\left[\mathrm{mmol}\cdot g^{-1}\right]=\frac{2\left[WL\left(T_{0}\right)-WL\left(T_{f}\right)\right]}{100\cdot M_{H2O}}$$

(3) $$n\left[OH\cdot m^{-2}\right]=\left(\frac{2\left[WL\left(T_{0}\right)-WL\left(T_{f}\right)\right]}{100\cdot M_{H2O}}\right)\left(\frac{N_{A}}{S_{BET}}\right)$$

where: WL(T_0_) − WL(T_f_) stands for mass loss (wt %) in the temperature range of interest: 110–230 °C, M_H2O_ is water molar mass, N_A_ is Avogadro constant, and S_BET_ is the specific surface area derived from BET.

It was found that the investigated samples were efficiently modified in the range of 81–96%. Considering the spacer molecules with the amino group attached and an increased alkyl chain length, i.e., C_4_H_8_NH_2_ (E_f_ = 96.2 %), and C_10_H_20_NH_2_ (E_f_ = 81.0 %), a slight reduction of the grafting efficiency was found. However, for the other types of spacer molecules, i.e., C_10_H_20_O_2_NH_2_ (ether linkage), the value of E_f_ was found to be over 85%. The established data were higher than the results presented in the literature for the grafting of alumina with the commercially available ATPES modifier, which was found in the range of 65–84 % [31,32,44].

The collected data for these samples are presented in Table 1. An increase of the hydrodynamic diameter of the functionalized samples was observed for the spacer molecules with longer chains. The same trend was found after enzyme immobilization (Table 1). Al_2_O_3_ powders modified with C_10_H_20_O_2_NH_2_ were found to however agglomerate more visibly than samples grafted with C_10_H_20_NH_2_. The above-mentioned observation can be linked to the chemical natures of the material and to the presence or absence of oxygen pendant groups. Furthermore, this trend may be related to a higher polydispersity index of the sample modified with C_10_H_20_O_2_NH_2_ (36%) than C_10_H_20_NH_2_ (23%). The polydispersity index increased with thicker organic layers grafted onto the ceramic materials. As an example, the functionalized ceramic with C_4_H_8_NH_2_ exhibited a polydispersity index of 12%, while for the C_10_H_20_NH_2_ it reached 23%.

Furthermore, the enzyme immobilization led to an increase of the polydispersity index from 25% to 31% for the C_4_H_8_NH_2_ and C_10_H_20_NH_2_, respectively. All of the functionalized ceramics possessed very good colloidal stabilities as confirmed by the distribution of the hydrodynamic diameters in water with near mono-dispersed distributions (Figure S14). The addition of enzymes however led to a reduction of the stability of the samples. The surface charge of the ceramic materials was monitored by zeta potential measurements in water (pH = 5.8). The surface charge was undoubtedly impacted by the functionalization process as well as by enzyme immobilization (Table 1). Although the method of enzyme anchoring had no influence on the zeta potential, a substantial change was observed on the electrophoretic mobility data acquired (Table 1). The lover value of the mobility, equal to 2.45 µm cm V^−1^ s^−1^ might be related to a higher loading of enzymes onto the particles due to the weak adsorption processes, and not only to covalent bonding. An interesting trend was found for chemical immobilization, since the electrophoretic mobility of the modified samples was reduced for longer spacer molecules chains. The values decreased from 3.18 to 3.03 µm cm V^−1^ s^−1^ for the C_4_H_8_NH_2_ + E and C_10_H_20_O_2_NH_2_ + E samples, respectively. As opposed to samples grafted with enzymes, the ceramic particles functionalized only with the spacer molecules were found to exhibit increasing electrophoretic mobility compared to those modified with longer spacer molcules. This trend is linked to greater degree of freedom of movement for the longer chains and upon enzyme addition, chains movement capabilities were strongly reduced.

The zeta potential values determined for the pristine alumina oxide was in a good accordance with literature data [45]. Zeta potential depends strongly on the particle size [46] and particles with a diameter smaller than 1 μm in aqueous solution generally carry a negative electric charge and have predisposition to approach one another under the Brownian movement rule [47]. According to the Derjaguin–Landau–Verwey–Overbeek (DLVO) theory [48], in a case when the kinetic energy of the colliding particles is high enough to overcome the barrier of energy from the electrostatic repulsive force, the van der Waals attractive force will prevail and cause the particles to agglomerate. This behavior supports coagulation or flocculation, thus increasing the size of colloidal aggregates over time [49]. In water, the stability of the nanoparticles and submicron-size particles are subject to strong electrostatic interactions or double-layer repulsive forces only if DLVO forces are present and the double-layer repulsive forces or electrostatic interactions are much stronger in this later case than van der Waals attractive forces [50]. For the range of non-DLVO attractive forces such as hydrophobic ones, bridging by adsorbed additives, patch charging, and hydrogen bonding of function groups of adsorbed additives will dominate the particle-particle interactions [51]. The cause of the substantial differences between zeta potential for the pristine, functionalized, and immobilized materials was therefore related to the addition of the spacer molecules and to the enzymes as well as likely to changes in the hydrophilicity/hydrophobicity patterns upon grafting.

**Figure 4:**
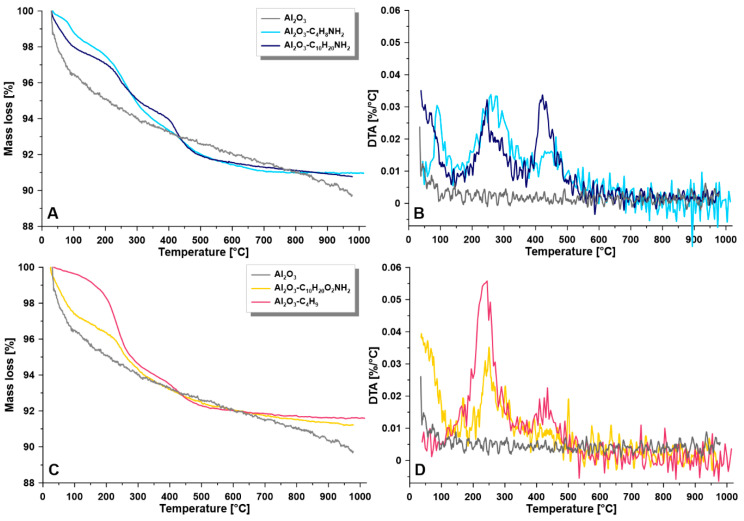
Thermal stability— thermogravimetric analysis (TGA) (**A**,**C**) and differential thermal analysis (DTA) (**B**,**D**) for the investigated samples; (**A**,**B**)—pristine sample and materials modified with C_4_H_8_NH_2_ and C_10_H_20_NH_2_; (**C**,**D**)—pristine and materials modified with C_10_H_20_O_2_NH_2_, C_4_H_9_.

TGA analyses were implemented to evaluate the thermal stability of the samples. For all modified samples, an improvement in thermal stability after functionalization with the spacer molecules was observed (Figure 4). The mentioned process was monitored by the temperature of decomposition of the coatings (T_d_). The decomposition process of the modifiers with shorter alkyl chains, i.e., C_4_H_8_NH_2_, and C_4_H_9_ were achieved in one step, at around 245 to 278 °C (Figure 4). Although for the pristine, ungrafted materials, the T_d_ was found at 253 °C, the highest T_d_ value was observed for the C_4_H_8_NH_2_ modified materials (T_d_ = 278°). This behavior can be explained by the efficiency of the grafting strategy and to the generation of well organized, densely packed materials with an organic layer anchored onto Al_2_O_3_ support. The longer spacer molecules however required more energy to decompose, and for that reason, the greater intensity of the peaks was found located at higher temperatures. In the case of grafting with C_10_H_20_NH_2_ two high peaks (ca. 0.033 %/°C) with the maxima at 263 and 420 °C were observed after primary thermal decomposition. The sample with the largest attached molecules of spacer molecules (C_10_H_20_O_2_NH_2_) required a greater input of energy to decompose the material. Two peaks at 251and 442 °C with respective values of 0.056%/°C and 0.023%/°C were found on the DTA curve for the sample C_10_H_20_O_2_NH_2_.

To summarize, the functionalization process of Al_2_O_3_ was characterized by high efficiency of grafting. The modification route was accomplished in the way which ensures the availability of terminal amine groups. The mentioned terminal groups (–NH_2_) will be crucial for the subsequent step of the process related to the enzyme immobilization.

#### 3.1.2. ZrO_2_ Ceramic Membranes

**Figure 5:**
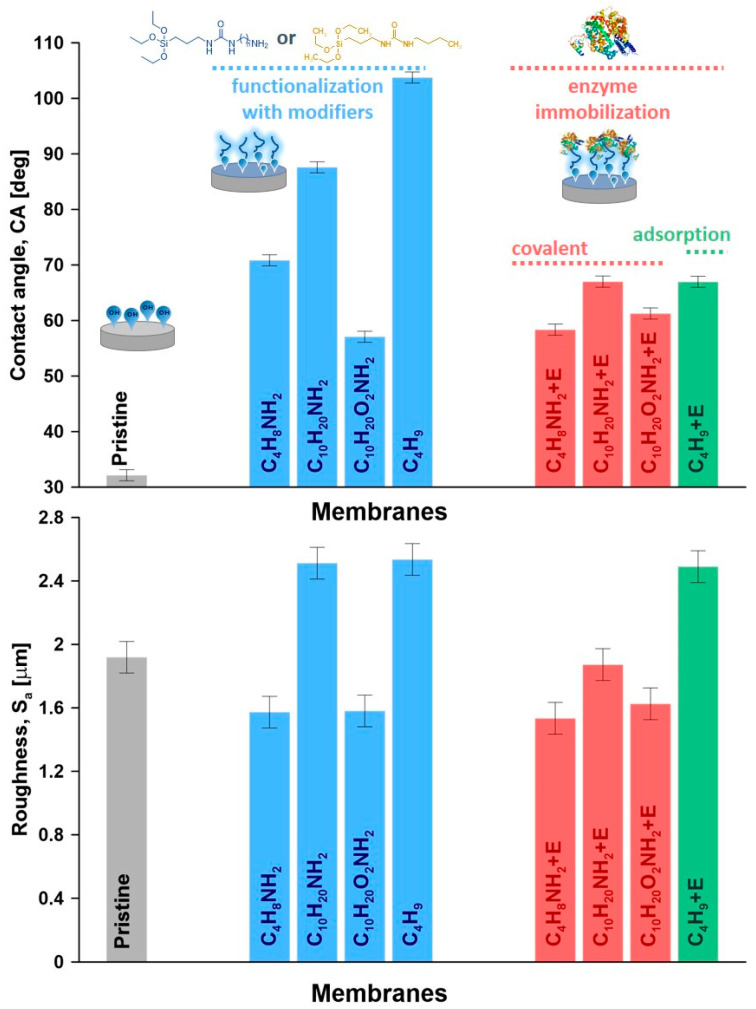
Apparent contact angle and roughness parameters (S_a_—arithmetic average) for the pristine, functionalized and immobilized materials.

The efficiency of the ceramic membrane functionalization by using modifiers with subsequent enzyme attachment was assessed by goniometric measurements coupled with topological analyses. The wettability of the materials provided an important information on how the modification and enzyme immobilization processes influence the material features (Figure 5 and Figure S14). As an effect of the functionalization process, an increase of apparent contact angle (Figure 5), as well as CA corrected by roughness (Figure S15), were observed. The surface of the pristine ceramic materials was turned from hydrophilic to hydrophobic upon grafting. The same trends were observed in our previous works focused on the functionalization of ceramic membranes for various membrane separation processes [31,52,53]. An increase in contact angle from 70.8° to 87.6° was observed with longer spacer chains, as expected from literature [54].

Subsequently, taking into account two modifiers with quite a similar length, i.e., C_10_H_20_NH_2_ and C_10_H_20_O_2_NH_2_, but different chemical compositions with and without oxygen pendant groups, significant changes were observed. The material functionalized with the spacer molecules containing oxygen (C_10_H_20_O_2_NH_2_) led to much lower contact angle of 57.1°. The explanation of such behavior can be found by taking inspiration from peptide chemistry [55,56]. Comparing the chemical structure of the newly synthesized modifiers in the presented research, it was possible to find the similarities with the amino acids and particularly with their side-chains. Taking into consideration the composition and length, the modifiers with the amine groups, i.e., C_4_H_8_NH_2_, C_10_H_20_NH_2,_ and C_10_H_20_O_2_NH_2_, it can be compared to the lysine or glycine, while the C_4_H_9_ modifier (without amine terminal group) can be compared to the isoleucine. Zhu and co-workers [57] developed a computational approach that can relate the molecular hydrophobicity scale of amino-acid chains to the contact angle of water nanodroplet.

The wettability of the lysine or glycine modified samples was found to be extremely high leading to instantaneous droplet disappearance, while for the isoleucine, the value remained stable at 121.8°. These values of contact angle previously reported in literature [57] shed light on a better explanation and understanding of the contact angle measured on ceramic membranes (Figure 5).

To analyze the wettability feature more in detail, after functionalization and then enzyme immobilization, the values of contact angle corrected by the roughness (CA_cor_), work of adhesion (W_adh_) and spreading pressure (S) for all of the investigated samples were determined. The gathered data are presented in Table 2. The corrected contact angle (CA_cor_) considered the influence of the roughness in the micro and macro-scale were found to be slightly higher than the apparent ones. This trend might be associated to the presence of micro-domains with hydrophobic character on the hydrophilic surface. On the other hand, the surface with contact angle below 90°, modified with C_4_H_9_ moiety, was characterized by a bit smaller value of CA_cor_ then apparent one. In this situation, the possibility of hydrophilic micro-areas could explain the observed alteration (Table 2). The work of adhesion and spreading pressure are very sensitive parameters in the assessment of physicochemical properties of modified materials. A smaller value of the W_adh_ indicates that weaker interactions between the material and water molecules were detected, corresponding directly with higher hydrophobicity. Indeed, the smallest value of adhesion work has been found for surface treated with C_4_H_9_ modifier. A slightly higher level of W_adh_ was observed for the ceramic materials after enzyme immobilization, ensuring the strongest interactions between enzyme rich surface and water molecules. Finally, the spreading pressure value that can be found close to zero when the surface is getting more hydrophilic was evaluated. Pristine sample, due to its nature, was characterized by the bigger value of the S, equal to –11.38 mN m^−1^. As an effect of the functionalization process, the spreading pressure reduced and was in the range of −33.46 and −90.03 mN m^−1^. After enzyme attachment to the surface of the particles by covalent bonding, the S values increased and ranged between −34.76 and −44.68 mN m^−1^.

### 3.2. Enzyme Immobilization

#### 3.2.1. Transesterification of (±)-1-Phenylethanol

**Figure 6:**
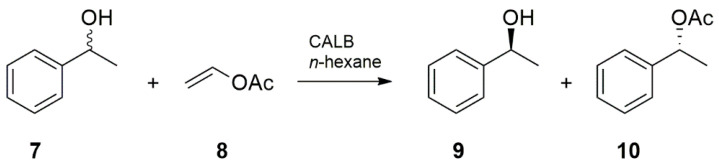
Kinetic resolution of (*R,S*)-1-phenylethanol catalyzed by *Candida antarctica* B lipase.

To characterize the catalytic performance of the immobilized lipase, transesterification of racemic (*R,S*)-1-phenylethanol **7** with vinyl acetate **8** to produce (*S*)-1-phenylethanol **9** and (*R*)-1-phenylethyl acetate **10** in *n*-hexane was selected as the test reaction (Figure 6).

The results of transesterification catalyzed by CALB immobilized on powders with various linker chain length are presented in Table 3.

As observed from these results, the eeP values in all studied samples were >95.5%. The enantiomeric ratio of the immobilized lipase was slightly lower in the case of a shorter linker chain. The results found for the samples modified with longer chains shows that the chemistry of the linkers has an important influence on the lipase loading. For the Al_2_O_3_ treated initially with spacers containing oxygen (C_10_H_20_O_2_NH_2_) the mass of loaded lipase was almost double than that for the C_10_H_20_NH_2_ one. The specific activity of the lipase expressed as the initial reaction rate per mass of the immobilized protein was dependent on the support and ranged from 0.31 to 1.2. As shown in Table 3, greater values of the mass reaction rate were obtained for supports functionalized with a longer spacer. The spacer molecules appear to have minimized the interactions between the enzyme and more hydrophilic membrane surfaces, which could affect the lipase activity, especially in reactions performed in organic media. This may result in higher values of substrate conversion and enantioselectivity. The above results give evidence that immobilization of lipase via spacer with elongated chain improves catalytic behavior of lipase.

#### 3.2.2. Transesterification of (±)-1-Phenylethanol in the Membrane Reactor

**Figure 7:**
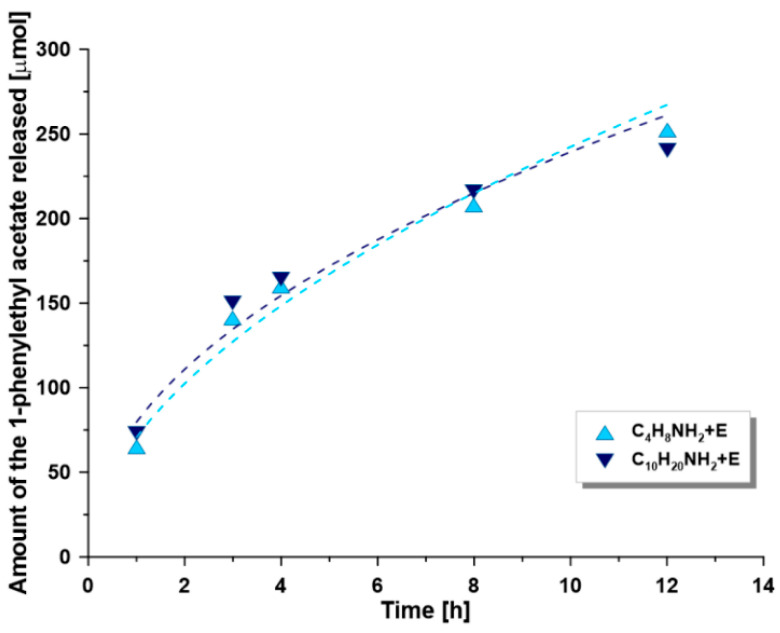
Amount of the 1-phenylethyl acetate produced vs. time for different CALB-immobilized membranes. (*R,S*)-1-phenylethanol 5 mM, 2-fold excess of vinyl acetate, *n*-hexane as a medium, temperature 30 °C.

The ZrO_2_ membranes with MWCO equal to 300 kDa were selected for the development of the biocatalytic membrane reactor due to the high surface area/volume ratio for enzyme loading and relatively high flow rates. CALB is made up of 317 amino acid residues with a formula weight of 33 273 Da [58], so that the membrane itself shall not retain the biocatalyst without its immobilization. Since the transesterification process is carried out in an organic phase (*n*-hexane), the ceramic membranes are relevant substrate materials choice, since resistant providing a stable platform for the reactions. The membrane acts here as a contactor and separator of the immobilized enzymes. The results of the transesterification of (*R,S*)-1-phenylethanol with vinyl acetate in the membrane reactor are provided in Table 4 and Figure 7.

The membranes modified with shorter spacer molecules were found to have higher enzyme loading and greater conversion ratio. The amounts of enzyme immobilized on the functionalized membranes varied in the range of 0.5 to 0.8 g of lipase per m^2^ of active area of membrane. Higher enzyme loadings for the shorter spacer molecules were probably related to the higher grafting efficiency for these compounds. However, the comparison of the specific activity, calculated as the ratio of initial reaction rate per mass of enzyme immobilized, shows an increase with respect to the length of the spacer molecule chain. This ratio is also higher than the values for the corresponding powders (Table 3), which suggests that the enzymes are there better distributed across the surface of the membranes than that observed onto the surface of powders. This trend may be related to the fact that the membranes provide easier access to the catalytic center of the enzyme since not prone to agglomeration. As a result, the enzyme was immobilized in excess which may negatively affect the absolute specific activity. This comment suggests that a larger number of enzymes does not necessarily relate to the reactor’s performance. The efficiency of the enzyme loading depends significantly on a number of other parameters including the type of linker, its chemical composition and length, as well as the type of the support surface (ceramic membrane or particles of oxide powder). Reaction profiles of transesterification of 1-phenylethanol with different lipase-immobilized membranes are shown in Figure 7.

As can be seen from Figure 7, the conversion was found to be higher in the reactor with membranes exhibiting higher enzyme loadings. The inter-samples variations are however relatively small and could be assumed to fall within experimental errors supporting the overall grafting strategy and the fact that the enzyme grafting was likely optimized for shorter spacer molecule chains length. The covalently lipase immobilized however retained a high level of enantioselectivity. The enantiomeric ratio for all the membranes was >200, and the enantiomeric excess of the product was ≥ 98% for a 30–45% total conversion. Although the observed values were higher than for corresponding functionalized alumina powders, it is impossible to determine the precise efficiency of grafting onto the membranes, and thus to compare the results directly. The cut-off and porosity of membranes may also play a significant role in the enzyme immobilization process and will be investigated in further research.

## 4. Conclusions

The generation of the hybrid materials with specifically designed spacer molecules, used to immobilize enzymes onto ceramic supports were successfully demonstrated. Four types of new spacer molecules with different length of alkyl chains as well as exhibiting various terminal groups were synthesized. The immobilization was performed onto two types of ceramic supports, i.e., Al_2_O_3_ powders and ZrO_2_ ceramic membranes. Very high efficiencies of grafting were reported (*ca.* 90%), which varied slightly based on the type of spacer molecules. An increase in length of the spacer molecules supported the generation of more hydrophobic materials with well-developed roughness. An element of novelty in this work was the demonstration of the contact angle corrected by roughness to gain exact information about the changes in wettability features. In the case of alumina powders, the enzyme loading was significantly improved upon grafting spacers with longer chains leading to greater grafting efficiencies. In addition, the specific activity of the samples was found to be much greater for samples functionalized with longer spacer molecules. Although the materials were found to exhibit great enantioselectivity (>97%), the enantiomeric ratio of the immobilized lipase was slightly lower in the case of the shorter spacer molecules. Overall, the efficiency of enzyme loading was found to depend on many factors, including type of spacer molecules, their chemical composition and length of the chains, as well as enzymes interactions with the surfaces of the supports.

## Figures and Tables

**Table 1:** Dynamic light scattering (DLS) and **ζ**—zeta potential characteristics of the ceramic materials.

	Particle Size [nm]	ζ Potential [mV]	Conductivity [µS cm^−1^]	Electrophoretic Mobility [µm cm V^−1^ s^−1^]
**Al_2_O_3_**	116	42.98	5.8	3.35
**Functionalization**				
**C_4_H_8_NH_2_**	289	25.64	6.9	2.00
**C_10_H_20_NH_2_**	540	42.04	2.8	3.28
**C_10_H_20_O_2_NH_2_**	376	42.31	2.4	3.30
**C_4_H_9_**	157	32.41	2.9	2.53
**Enzyme Immobilization**				
**C_4_H_8_NH_2_ + E**	493	40.87	6.0	3.18
**C_10_H_20_NH_2_ + E**	608	39.70	2.6	3.03
**C_10_H_20_O_2_NH_2_ + E**	452	38.93	2.8	3.03
**C_4_H_9_ + E**	193	31.53	2.6	2.45

**Table 2:** Wettability study of the investigated samples.

	CA_cor_ (deg)	W_adh_ (mN m^−1^)	S (mN m^−1^)
**Pristine**	32.5 ± 1.5	134.2 ± 2.2	−11.38 ± 0.23
Functionalization			
**organic spacer**			
**C_4_H_8_NH_2_**	71.0 ± 1.5	96.5 ± 1.4	−49.14 ± 0.36
**C_10_H_20_NH_2_**	86.9 ± 1.5	76.7 ± 1.7	−68.88 ± 0.41
**C_10_H_20_O_2_NH_2_**	57.3 ± 1.5	112.1 ± 2.3	−33.46 ± 0.28
**C_4_H_9_**	103.7± 1.5	55.6 ± 1.3	−90.03 ± 0.48
Enzyme Immobilization			
**C_4_H_8_NH_2_ + E**	58.5 ± 1.5	110.8 ± 2.2	−34.76 ± 0.33
**C_10_H_20_NH_2_ + E**	67.3 ± 1.5	100.9 ± 2.0	−44.68 ± 0.39
**C_10_H_20_O_2_NH_2_ + E**	61.4 ± 1.5	107.6 ± 2.2	−37.97 ± 0.35
**C_4_H_9_ + E**	67.1 ± 1.5	102.5 ± 1.9	−43.14 ± 0.41

**Table 3:** Enzyme load and results of transesterification of (*R,S*)-1-phenylethanol with vinyl acetate catalyzed by CALB immobilized on modified powders. Enzyme immobilization strategy—covalent bonding—C_4_H_8_NH_2_ + E, C_10_H_20_NH_2_ + E, C_10_H_20_O_2_NH_2_ + E; physical adsorption—C_4_H_9_ + E.

Powder	Mass of lipase per mass of support (mg g^−1^)	Specific activity (μmol min^−1^ mg^−1^)	Conversion * (%)	eeS (%)	eeP (%)	E **
**C_4_H_8_NH_2_ + E**	6.12	0.22	30	42	98	120
**C_10_H_20_NH_2_ + E**	7.83	0.81	42	71	99	>200
**C_10_H_20_O_2_NH_2_ + E**	13.85	1.20	41	68.5	99	>200
**C_4_H_9_ + E**	15.28	4.25	34 ***	49	95.5	65

* at 12 h, ** Enantiomeric ratio calculated according to Chen et al. [40], *** at 6 h, eeS—enantiomeric excess of the substrate; eeP—enantiomeric excess of the product; E—enantioselectivity.

**Table 4:** *Candida antarctica* type B (CALB) lipase immobilized membranes’ performance in transesterification of (*R,S*)-1-phenylethanol with vinyl acetate. Enzyme immobilization strategy—covalent bonding—C_4_H_8_NH_2_ + E, C_10_H_20_NH_2_ + E, C_10_H_20_O_2_NH_2_ + E; physical adsorption—C_4_H_9_ + E.

Membrane	Mass of lipase per membrane area (g m^−2^)	Specific activity (μmol min^−1^ mg^−1^)	Conversion * (%)	eeS (%)	eeP (%)	E **
**C_4_H_8_NH_2_ + E**	0.772	0.96	40	66	98	>200
**C_10_H_20_NH_2_ + E**	0.503	1.67	32	46.5	99	>200
**C_10_H_20_O_2_NH_2_ + E**	0.672	1.61	37	58	99	>200
**C_4_H_9_ + E**	1.494	1.19	44	74	95	78

* at 48h, ** Enantiomeric ratio calculated according to Chen et al. [40].

## Data Availability

Not applicable.

## Supplementary Materials

The following are available online at https://www.mdpi.com/1996-1944/14/1/201/s1, Figure S1. Fringe Projection Phase-Shifting schematics (A) and an example of a surface with the projected pattern (B). A sinusoidal pattern is sequentially projected on the sample surface and a camera is utilized to capture the fringe patterns and reconstruct the 3D image by phase-shift coding. Figure S2. In the sinusoidal phase-shifting method a series of phase-shifted sinusoidal patterns are recorded (top), from which the phase information at every pixel is obtained (bottom). The phase shift correlates with sample surface topography from pixel to the pixel that defines the resolution. Figure S3. The test synthesis of 1-butyl-3-(3-triethoxysilylpropyl)urea (3). Figure S4. 1H NMR spectra of 3. Figure S5. 13C NMR spectra of 3. Figure S6. IR spectrum of 3. Figure S7. Synthesis of modifiers and attachment way of the modifier molecules to the ceramic support, i.e., Al_2_O_3_ powders or ceramic membranes. Figure S8. A cross-flow reactor for ceramic membrane modification. Figure S9. Scheme of covalent immobilization. Figure S10. ATR-FTIR spectra of pristine and modified Al_2_O_3_. A and B parts are presented in Figure 3 in detail. Figure S11. Range of ATR-FTIR spectra representing the attachment of modifiers to the surface. Spectra of pristine and modified Al_2_O_3_. Figure S12. HR-TEM—bright field (A), SEAD images (B) and EDX spectra (C). Pristine Al_2_O_3_. Figure S13. HR-TEM—bright field (A,D), SEAD images (B,E) and EDX spectra (C,F). A–C—sample modified with C_10_H_20_O_2_NH_2_, D–F—sample functionalized with C_10_H_20_O_2_NH_2_ and enzymes. Figure S14. Changes in hydrodynamic diameter determined by DLS after modification with C_4_H_8_NH_2_ and enzyme immobilization. Figure S15. Correlation between contact angle and roughness of the surface.
