# Supplementary material for: Molecular Decoration of Ceramic Supports for Highly Effective Enzyme Immobilization—Material Approach
Source: Materials (Basel). 2021 Jan 3;14(1):201. doi: 10.3390/ma14010201 (PMC7794798; doi:10.3390/ma14010201)
Supplement: Supplementary file 1 [file materials-14-00201-s001.pdf]

materials

Article

Supplementary Materials: Molecular Decoration of Ceramic Supports for Highly Effective Enzyme Immobilization—Material Approach

Joanna Kujawa [1], Marta Głodek [1], Izabela Koter [1], Borys Ośmiałowski [1], Katarzyna Knozowska [1], Samer Al-Gharabli [2], Ludovic F. Dumée [3,4,5] and Wojciech Kujawski [1,*]

[1] Faculty of Chemistry, Nicolaus Copernicus University in Toruń, 7 Gagarina Street, 87-100 Toruń, Poland; joanna.kujawa@umk.pl (J.K.); marta.glodek1@umk.pl (M.G.); ikoter@umk.pl (I.K.); borys.osmialowski@umk.pl (B.O.); katkno@doktorant.umk.pl (K.K.)
[2] Pharmaceutical and Chemical Engineering Department, German-Jordanian University, Amman 11180, Jordan; samer.gharabli@gju.edu.jo
[3] Institute for Frontier Materials, Deakin University, Geelong, Waurn Ponds, Victoria 3216, Australia; ludovic.dumee@ku.ac.ae
[4] Center for Membrane and Advanced Water Technology, Khalifa University, P.O. Box 127788, Abu Dhabi, Saudi Arabia
[5] Department of Chemical Engineering, Khalifa University, Abu Dhabi, P.O. Box 127788, Saudi Arabia
* Correspondence: wkujawski@umk.pl; Tel.: +48-56-611-45-17

Citation: Kujawa, J.; Głodek, M.; Koter, I.; Ośmiałowski, B.; Knozowska, K.; Al-Gharabli, S.; Dumée, L.F.; Kujawski, W. Molecular Decoration of Ceramic Supports for Highly Effective Enzyme Immobilization—Material Approach. *Materials* **2021**, *14*, x. https://doi.org/10.3390/xxxxx

Received: 12 December 2020
Accepted: 29 December 2020
Published: 3 January 2021

Publisher's Note: MDPI stays neutral with regard to jurisdictional claims in published maps and institutional affiliations.

Copyright: © 2021 by the authors. Licensee MDPI, Basel, Switzerland. This article is an open access article distributed under the terms and conditions of the Creative Commons Attribution (CC BY) license (http://creativecommons.org/licenses/by/4.0/).

S1. Experimental part – the Fringe Projection Phase-Shifting

The contact angle corrected by roughness it is the value of the contact angle on the Young surface. An implementation of the fringe projection phase-shifting method is giving a unique opportunity to analyze surface roughness and contact angle of the same location of the sample. As a result, the contact angle on the rough surface, flat surface, roughness, and area factor of roughness are determined. In Fig. S1 and Fig. S2 the principal of the method is presented.

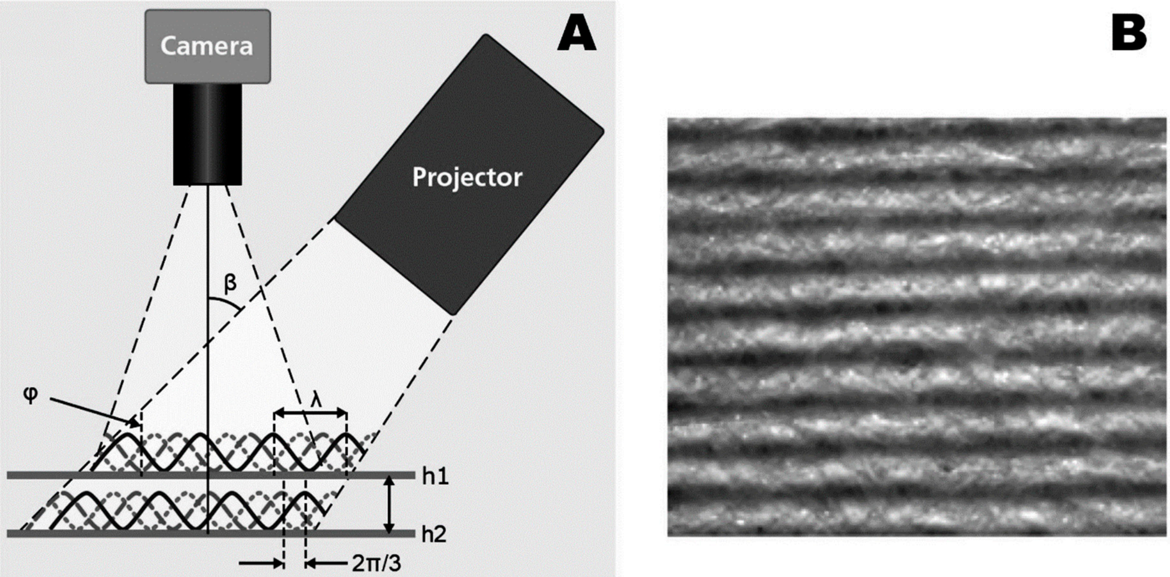

Figure S1. Fringe Projection Phase-Shifting schematics (**A**) and an example of a surface with the projected pattern (**B**). A sinusoidal pattern is sequentially projected on the sample surface and a camera is utilized to capture the fringe patterns and reconstruct the 3D image by phase-shift coding.

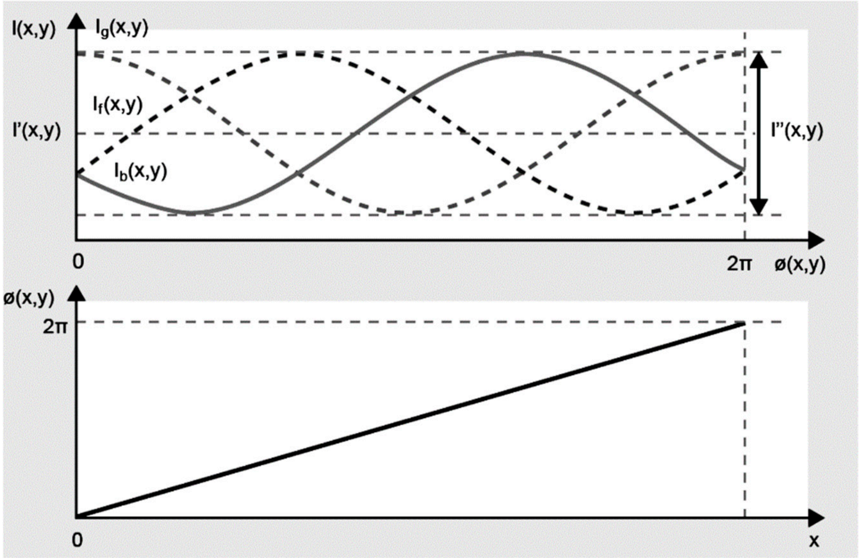

Figure S2. In the sinusoidal phase-shifting method a series of phase-shifted sinusoidal patterns are recorded (top), from which the phase information at every pixel is obtained (bottom). The phase shift correlates with sample surface topography from pixel to the pixel that defines the resolution.

As illustrated in Figure S1 the 3D topography module consists of a projector with an LED light source and a slide with sinusoidal fringe patterns on a grey-scale. These illumination patterns are sequentially projected onto the studied surface, and a digital camera captures the fringe patterns from which the 3D shape of the object is reconstructed by phase-shift coding. This enables pixel level measurement resolution. In the 3D Topography Module, the pixel size is 1.1 μm x 1.1 μm for analysis of micron-scale surface features.

The sinusoidal fringes can be expressed by

$$I_n(x, y) = a + b\cos\left(\frac{2px}{p} + \varphi_0 + \delta_n\right) \quad (1)$$

where (x, y) is the coordinate in the slide frame plane, a is background intensity, b is amplitude modulation, p is the sinusoidal grating wavelength, φ_0 is the additional phase shift caused by the surface height and δ_n is the phase shift from the slide movement.

As an example, for a color pattern with red, green, and blue light, it can be demonstrated the case of three divided wavelengths where I_r, I_g, and I_b are the corresponding intensities for each of the colors. The phase shifts can be plotted as in Figure S2.

Then the spatial phase shift can be expressed with the following equation

$$\varphi(x, y) = \arctan\left|\sqrt{\frac{I_r - I_b}{2I_g - I_r - I_b}}\right| \quad (2)$$

The phase shift indicates the horizontal coordinate, i.e. the height differences in every pixel providing the sample topography.

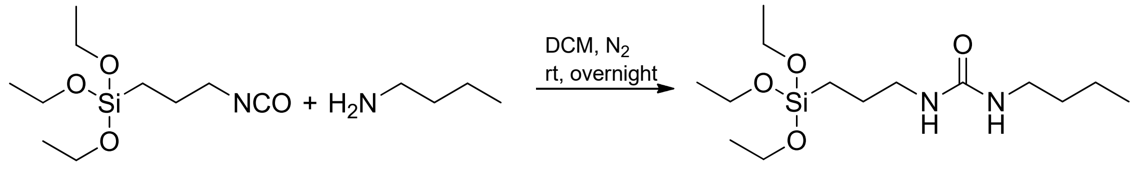

Figure S3. The test synthesis of 1-butyl-3-(3-triethoxysilylpropyl)urea (**3**).

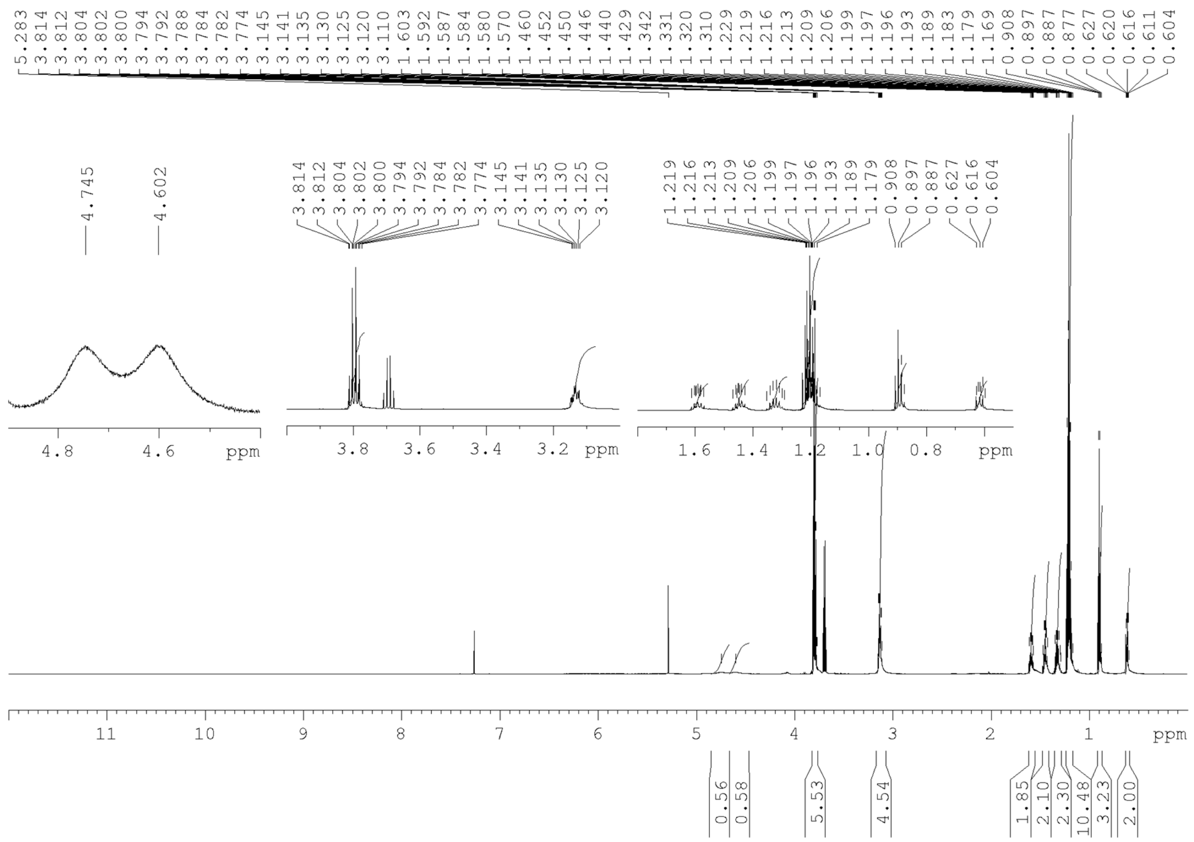

Figure S4. ^{1}H NMR spectra of **3**.

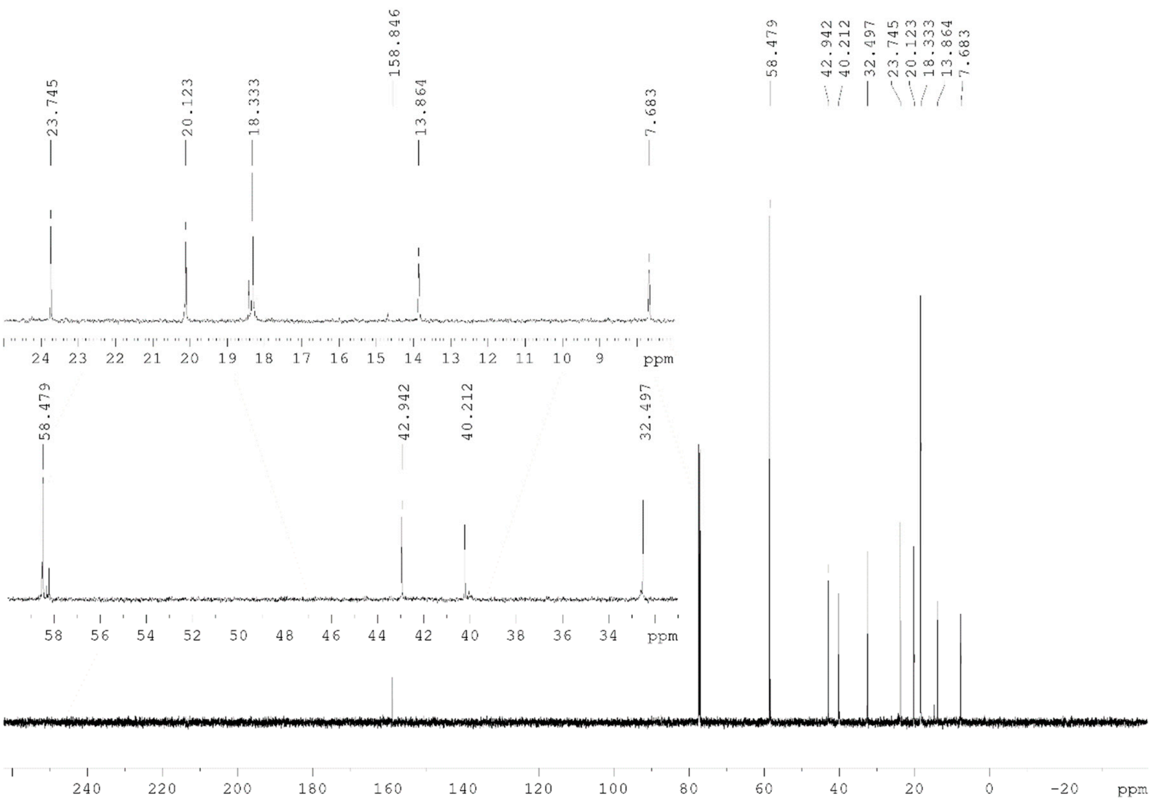

Figure S5. ^{13}C NMR spectra of **3.**

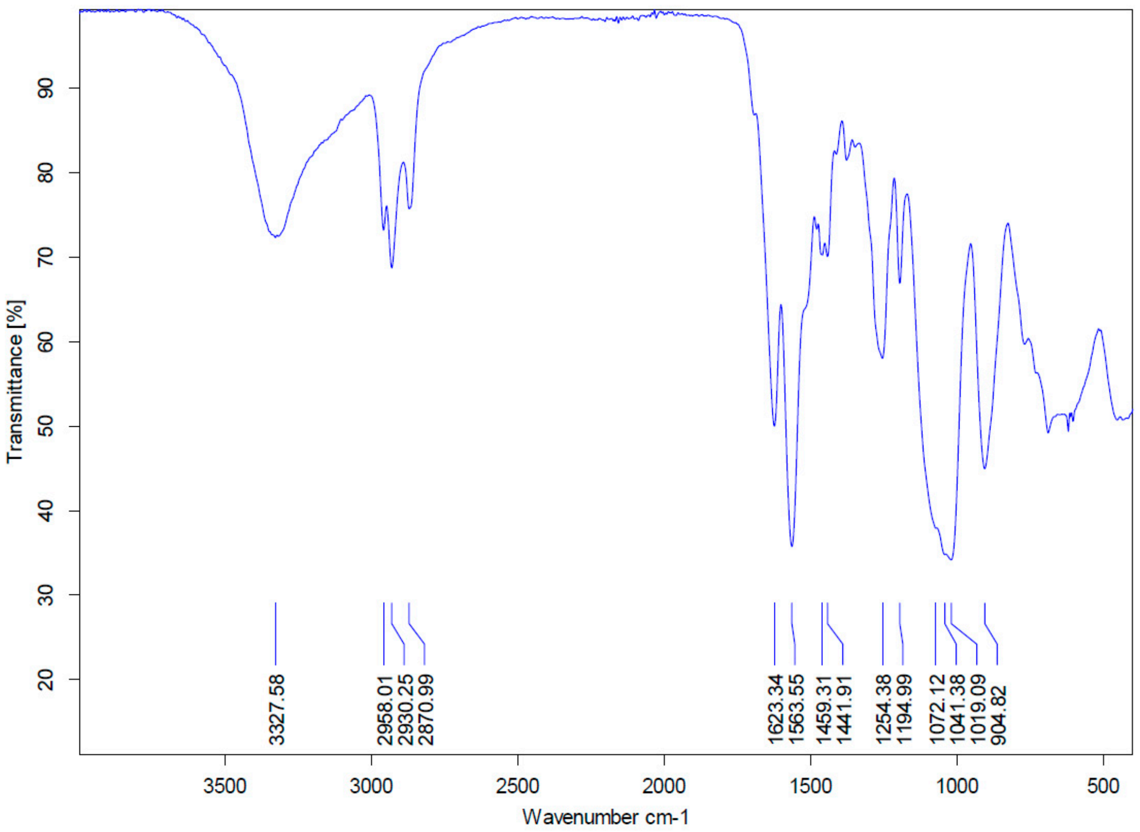

Figure S6. IR spectrum of **3.**

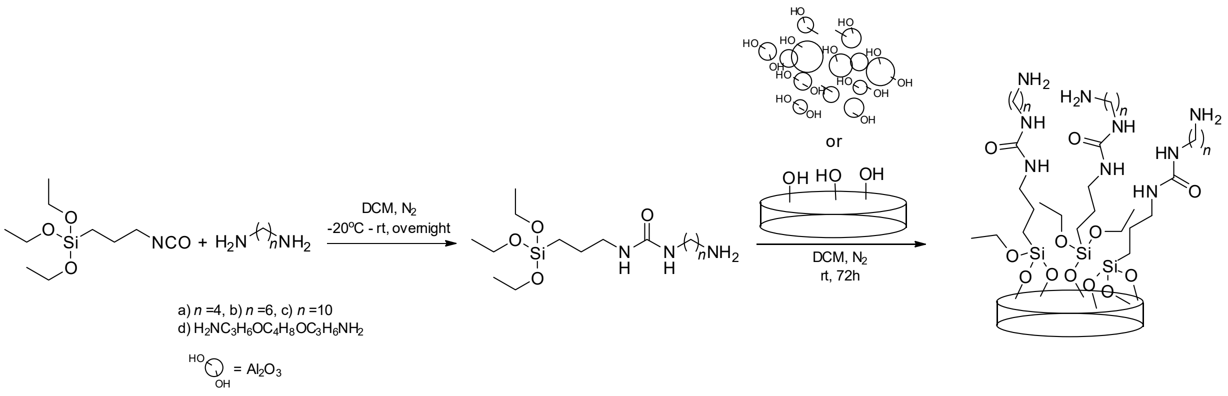

1 **4a-d** **5a-d** **6a-d**

Figure S7. Synthesis of modifiers and attachment way of the modifier molecules to the ceramic support, i.e. metal oxide powders or ceramic membranes.

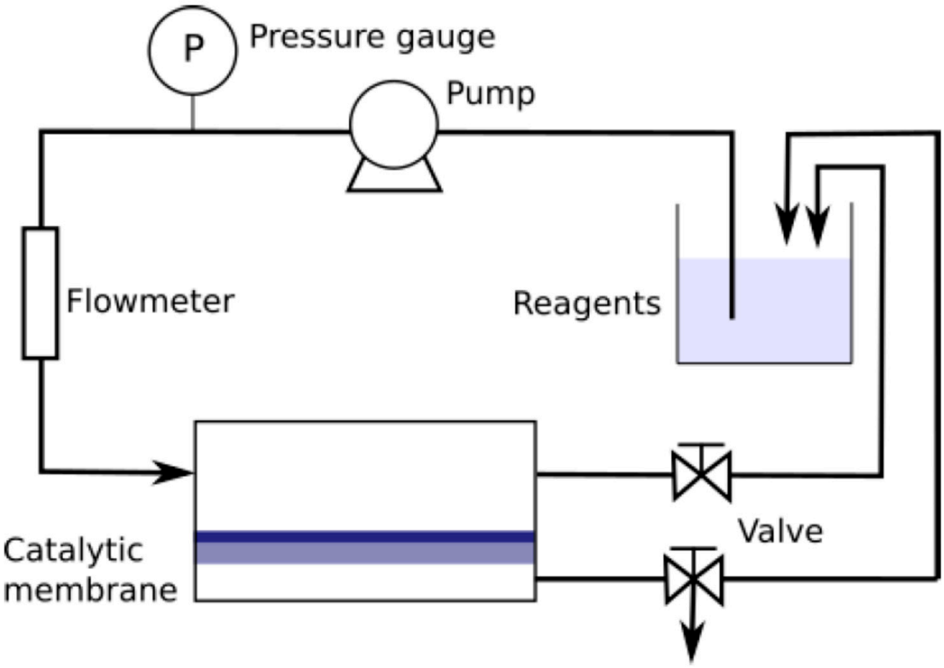

Figure S8. A cross-flow reactor for ceramic membrane modification.

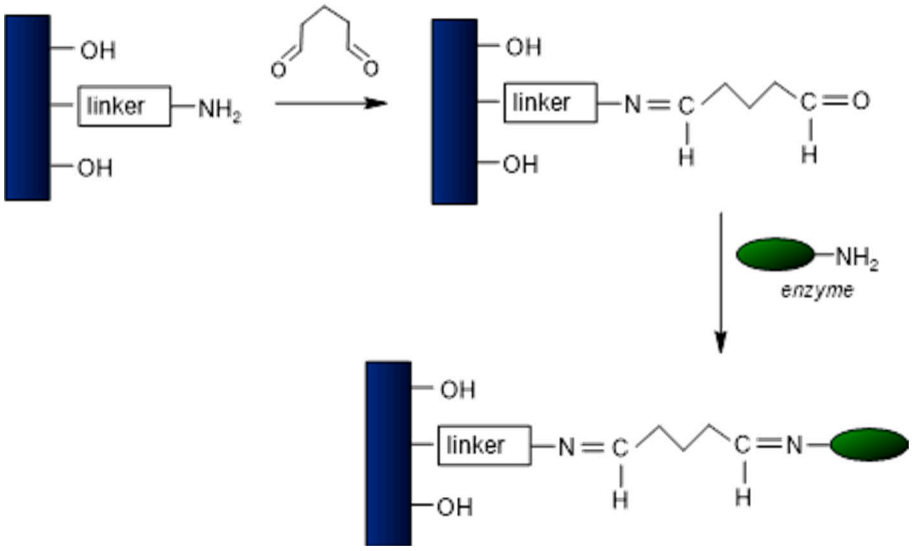

Figure S9. Scheme of covalent immobilization.

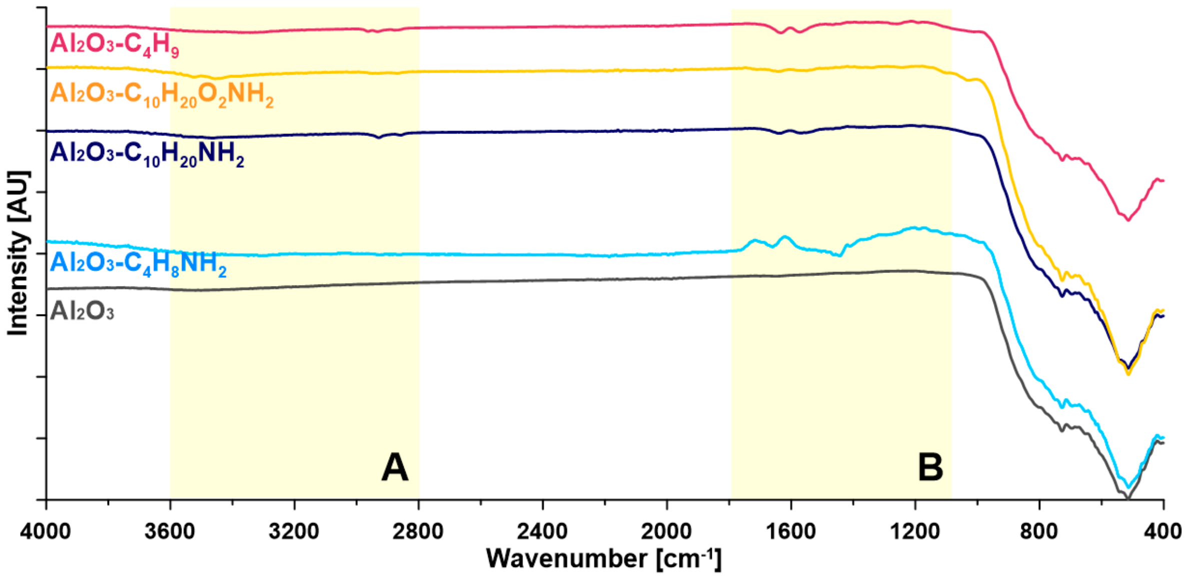

Figure S10. ATR-FTIR spectra of pristine and modified metal oxide powders. A and B parts are presented in Fig. 3 in detail.

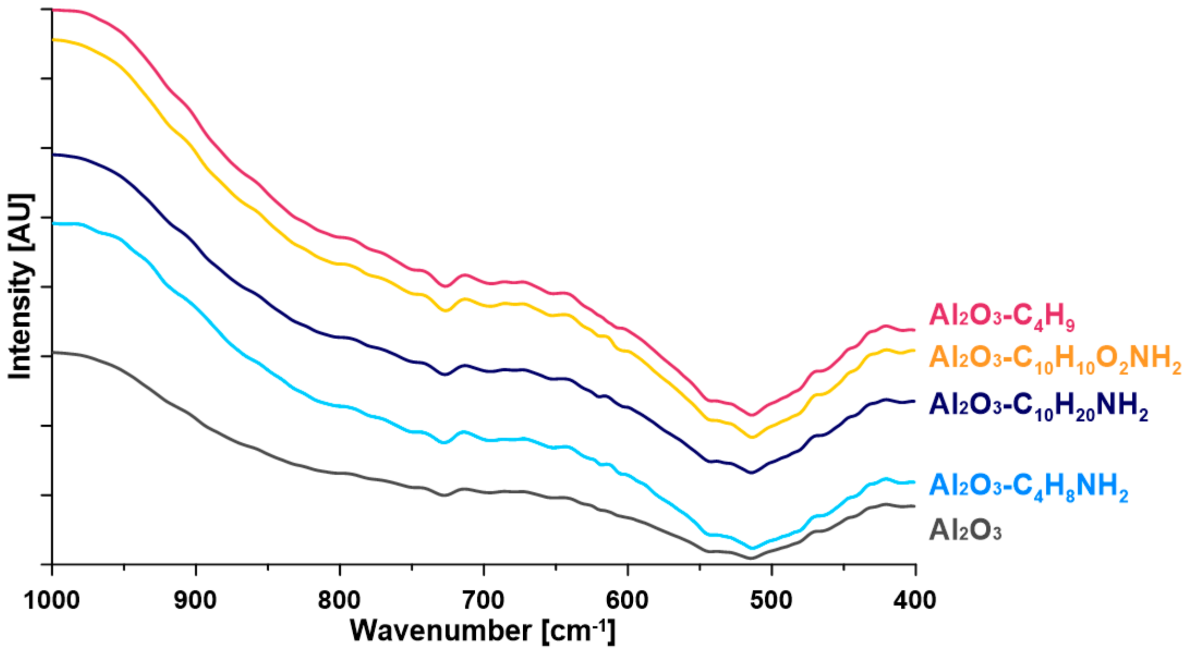

Figure S11. Range of ATR-FTIR spectra representing the attachment of modifiers to the surface. Spectra of pristine and modified metal oxide powders.

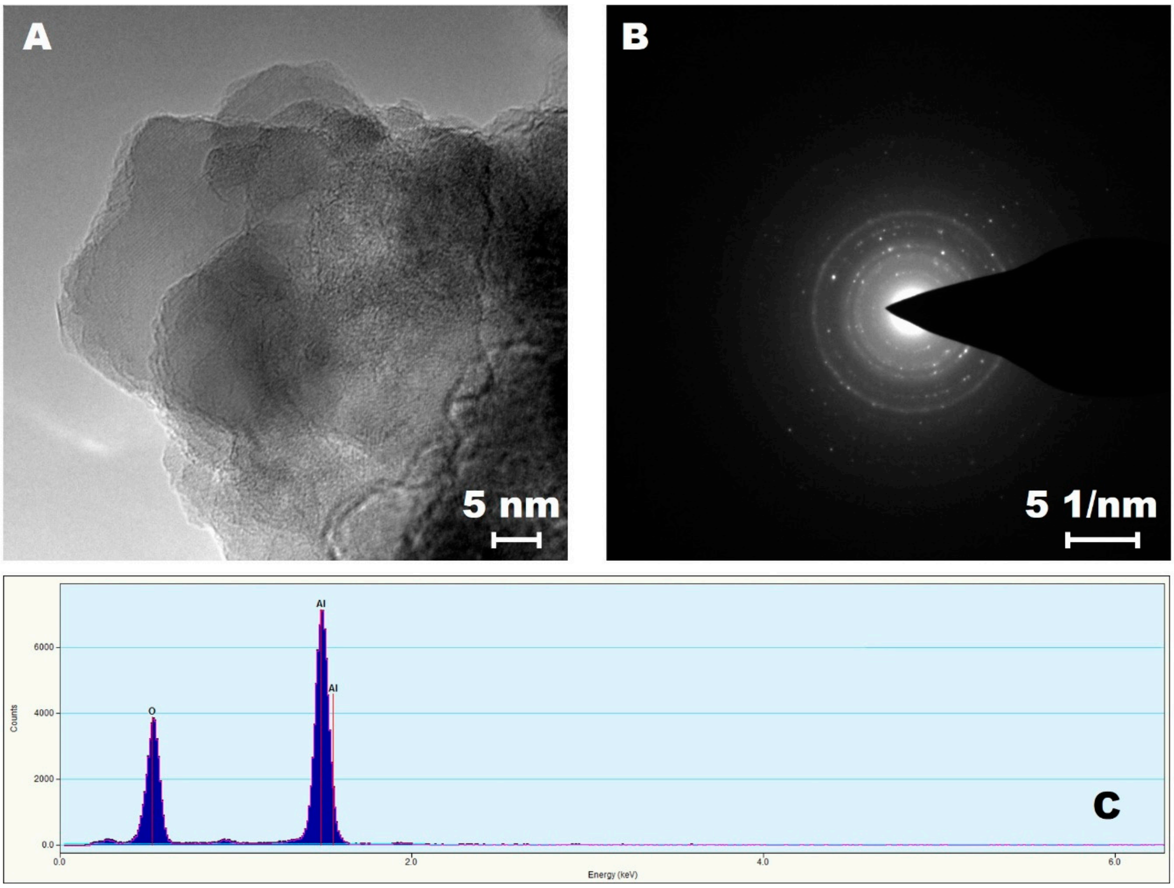

Figure S12. HR-TEM – bright field (**A**), SEAD images (**B**) and EDX spectra (**C**). Pristine Al_2O_3.

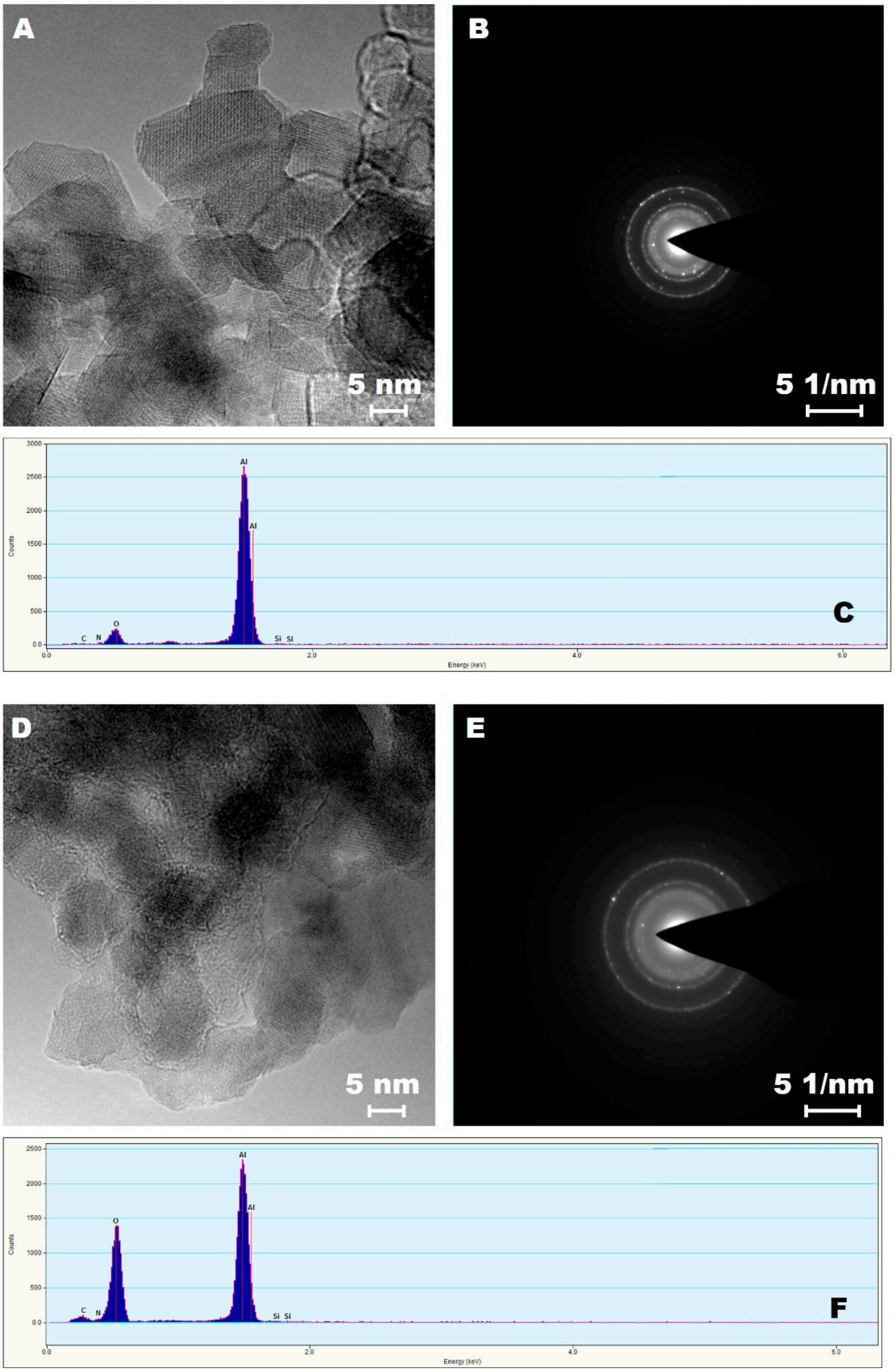

Figure S13. HR-TEM – bright field (**A**,**D**), SEAD images (**B**,**E**) and EDX spectra (**C**,**F**). A, B, C – sample modified with $C_{10}H_{20}O_2NH_2$, D, E, F - sample functionalized with $C_{10}H_{20}O_2NH_2$ and enzymes.

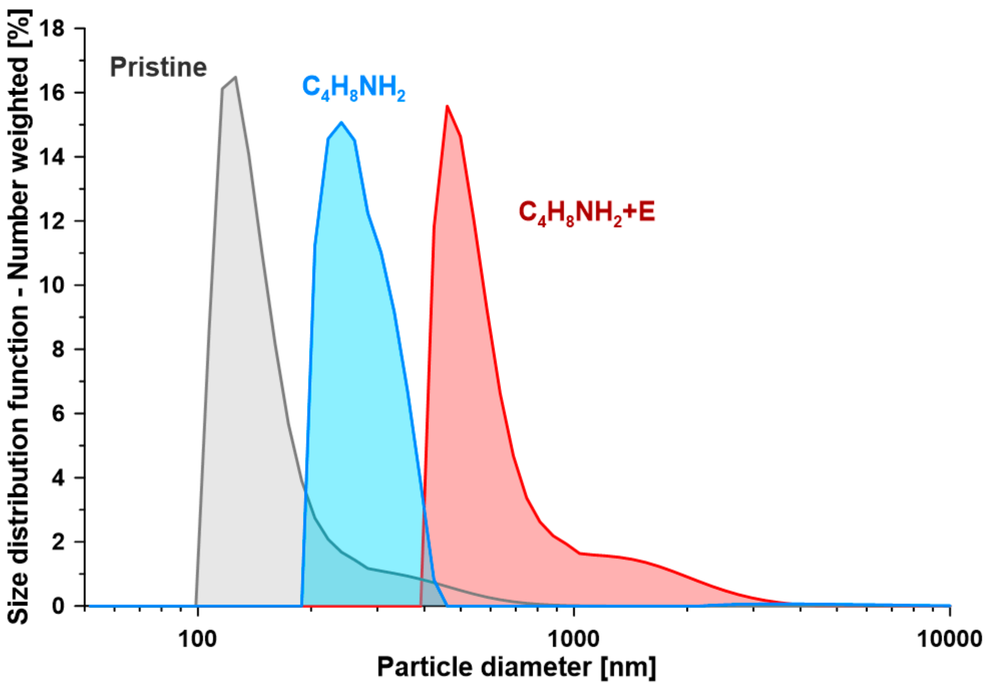

Figure S14. Changes in hydrodynamic diameter determined by DLS after modification with $C_4H_8NH_2$ and enzyme immobilization.

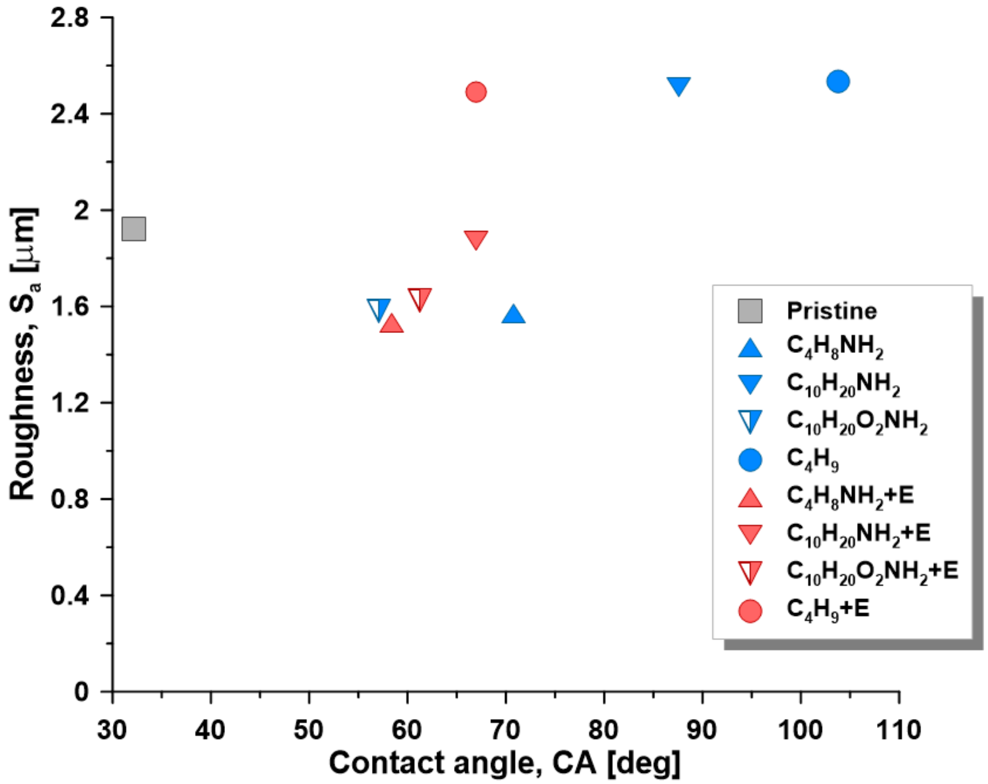

Figure S15. Correlation between contact angle and roughness of the surface.
